# Supplementary figures and images for: Diacerein provokes apoptosis, improves redox balance, and downregulates PCNA and TNF-α in a rat model of testosterone-induced benign prostatic hyperplasia: A new non-invasive approach
Source: PLoS One. 2023 Nov 9;18(11):e0293682. doi: 10.1371/journal.pone.0293682 (PMC10635502; doi:10.1371/journal.pone.0293682)

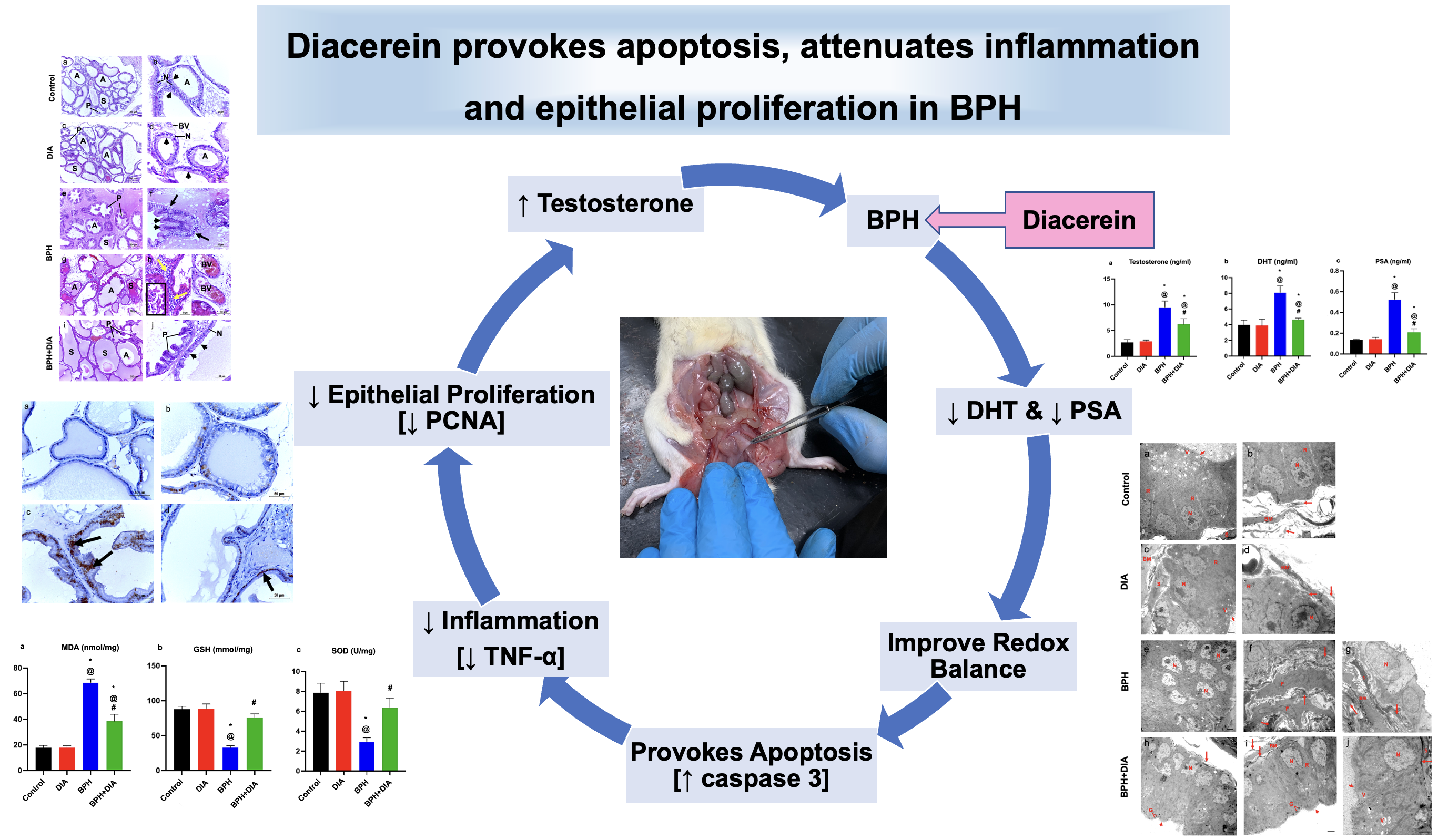

Supplement: S1 Graphical abstract — (TIFF) [file pone.0293682.s001.tiff]
